# Effectiveness of infrastructural interventions to improve access to safe drinking water in Latin America and the Caribbean on the burden of diarrhoea in children <5 years: a systematic literature review and narrative synthesis

**DOI:** 10.1080/16549716.2025.2451610

**Published:** 2025-02-14

**Authors:** Philippa Redondo, Tuba Mazhari, Amal R. Khanolkar

**Affiliations:** a Faculty of Life Sciences & Medicine, King’s College London, London, UK; b Department of Population Health Sciences, School of Life Course & Population Sciences, Guy’s Campus, King’s College London, UK

**Keywords:** Latin America and the Caribbean, access, drinking water, child health, diarrhoea

## Abstract

Globally, Latin America and the Caribbean (LAC) has one of the lowest rates of equitable access to safely managed drinking water. This systematic literature review assessed the effectiveness of infrastructure interventions to provide equitable access to safely managed drinking water in LAC on the burden of diarrhoea in children <5 years. The review was conducted in February 2024 using Ovid MEDLINE, Embase, Global Health, and the Cochrane Library with inclusion criteria: quantitative study designs of intervention effectiveness on burden of diarrhoea in children; conducted in LAC; studies published since 1 January 2000; and full-text available in English. Study quality was assessed via the US Agency for Healthcare Research and Quality scale. Reported quantitative data for diarrhoea burden of disease were extracted, and thematic analysis informed a narrative synthesis. Six studies from three countries in LAC with >110,000 data-points were included. Water supply infrastructure interventions were effective at reducing the burden of diarrhoea in children <5 years. Household level, rather than community level, access to a piped water supply, a continuous reliable service with <1 day of service interruption per month, and cash transfer programs for environmental public health programs, were identified as key contributors to water infrastructure intervention effectiveness. Previous water supply infrastructure interventions which include the provision of a safe drinking water supply are effective in reducing burden of diarrhoea in children. Future studies are needed to develop a comprehensive understanding of the unique features which contribute to water infrastructure effectiveness.

## Paper context

- **Main findings:** In Latin America and the Caribbean, water infrastructure interventions that promote improved population access to safely managed water supply are effective at reducing the burden of diarrhoea in children <5 years.
- **Added knowledge:** This study indicates that household level, rather than community level, access to a piped water supply, a continuous reliable service with <1 day of service interruption per month, and cash transfer programs for environmental public health programs, are key contributing features of water infrastructure intervention effectiveness.
- **Global health impact for policy and action:** There is limited knowledge on the required aspects of water supply infrastructure to ensure safe drinking water across Latin America and the Caribbean, highlighting the urgent need for further research and targeted public health interventions.

## Background

Increased climate change-induced water insecurity (i.e. inequitable access to clean, safe, and affordable water for consumption) is associated with an increased risk of water-related diseases [1–4]. Globally, the fourth leading cause of death in children <5 years is diarrhoea with 500,664 deaths in 2019, and 70% of these are directly attributable to unsafe water sources [5–7]. The United Nations (UN) Sustainable Development Goals target 6.1 outlines the goal to ‘*achieve universal and equitable access to safe and affordable drinking water for all*’ by 2030 [8–10]. There is considerable global and regional variation in access to safe and affordable drinking water, as defined by the proportion using reliable uncontaminated on-site water services [9,11,12].

**Figure 1.**
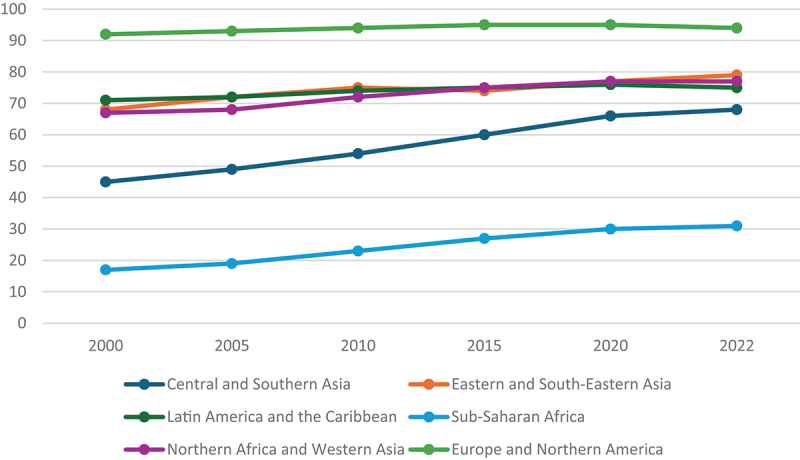
Population proportion using safely managed drinking water by region, 2000–2022 (%).

Latin America and the Caribbean (LAC), Central and Southern Asia, and Sub-Saharan Africa have the lowest rates of equitable access to safely managed drinking water at 75%, 68% and 31% in 2022, respectively, compared to 94% in Europe and North America [12]. Globally, rates of access to safely managed drinking water have shown an increasing trend; however, in LAC there has been a smaller increase from 71% in 2000 to 75% in 2022 (Figure 1) [12,13]. The plateau in the rate of access to safely managed drinking water in LAC may be attributable to the rapid urban growth, water scarcity as a result of climate change, and governance and economic challenges [14,15].

Due to a lack of access to safely managed drinking water, particularly in vulnerable populations such as children <5 years, there is an increased risk in morbidity and mortality from diarrhoea, with estimates suggesting 395,000 avoidable deaths each year globally [16–18]. In LAC, diarrhoea is the seventh leading cause of death in children <5 years [7], and there is an increasing proportion which results from septic bacterial infection [19]. The most common pathogens causing diarrhoea in children are rotavirus, norovirus, adenovirus, and astrovirus; with diarrhoea commonly resulting in malnutrition in children which leaves them at greater risk from adverse outcomes associated with diarrhoeal disease [19]. Common methods for prevention of diarrhoeal disease in children include safe drinking water and improved sanitation [19]. In LAC, the main drivers of unsafe water include insufficient management of water resources, inequity of access, climate change, and chemical contamination [9,20–22]. Therefore, it is important to assess the effectiveness of water supply infrastructure developments, such as increasing the number of households with access to piped water, to improve access to safely managed drinking water [9,20–22].

Existing systematic literature reviews (SLRs) highlight limited evidence on strategies to increase access to safely managed drinking water supplies and the effectiveness on improving health outcomes in children [23–26]. Given the outlined evidence gap and persistent global health challenge, this SLR aimed to report the effectiveness of infrastructure interventions to provide equitable access to safely managed drinking water in LAC on the burden of diarrhoea in children <5 years.

## Methods

This SLR was conducted according to the Preferred Reporting Items for Systematic Reviews and Meta-Analysis (PRISMA) guidelines, shown in Supplementary Appendix 1 [27].

### Search strategy

A literature search was conducted in February 2024 using a search strategy tailored for each of the chosen databases: Ovid Medline, EMBASE, Global Health, and the Cochrane Library. The full search terms by database are reported in Supplementary Appendix 2, which includes the use of a previously developed publicly available search strategy for the geographic region of the Caribbean [28]. The inclusion criteria included: studies (observational, cohort, crosssectional, ecological, or randomised controlled trial study designs) published since 1 January 2000, to capture studies immediately prior and since the introduction of the millennium development goals; investigation of water infrastructure intervention effectiveness; reporting quantitative burden of diarrhoea outcome data, including prevalence, morbidity, or mortality, for children <5 years; conducted in LAC; and full-text available in English. Studies were excluded if they did not meet any of the above criteria. One researcher conducted the search and selected the included studies independently, supervised by a second researcher.

### Data extraction

Key study characteristics were extracted from all selected studies and included study author and year, country, study design, aim, study population/sample, and methodology (Table 1).

**Table 1.** Extracted study characteristics from studies included in the systematic review.

Study	Country	Study Design	Aim	Population/ Data Source	Study Sample
Calzada et al. *World Dev.*, 2021	Peru	Cross-sectional	To assess the performance of the JASS* vs. public provision with respect to the impact on child health	Demographic and Health Survey, 2010–2014 Census data and household interviews with mothers of children <age 5	Pooled cross-section of 84,600+ households and 26,200 children <age 5
de Souza et al. *BMC Public Health*, 2021	Brazil	Ecological	To assess the interactive effects of public health interventions, environmental health programs, and a Conditional Cash Transfer Program (PBF), on reduction in mortality by diarrhoea	National registries, 2006–2016	3467 municipalities from all regions; 38137 observations of children <age 5
de Souza et al., *PLoS ONE*, 2021	Brazil	Ecological (longitudinal analysis)	To evaluate the interactive effects of improvements in water access with a Conditional Cash Transfer Program (PBF) on reduction in morbidity by diarrhoea	National registries, 2006–2016	3467 municipalities from all regions; 38137 observations of children <age 5
Hubbard et al. *J. Hyg. Environ*., 2011	Peru	Ecological observational	To describe the strategic approach and implementation of a condominial water and sanitation service	Population of Manuel Cardozo Davila settlement; Project implementation and health surveillance	680 households with children <age 5
Huicho et al. *J. Glob. Health*, 2019	Peru	Country case study	Document national trends (1980 t0 2015) in mortality from diarrhoea, interventions and policies aimed at diarrhoea	National population; Desk reviews, dataset analysis, and interviews; 1980–2015	NR; Children <age 5
Trudeau et al. *IJPH*, 2018	Guatemala	Cross-sectional	To examine effect of water system unreliability on incidence of diarrhoea	Nationally representative survey of households; Secondary data analysis	7579 children from households with children <age 5

*JASS= Juntas Administrativas de Servicios de Saneamiento (community-based water system). NR = Not Reported.

### Quality assessment

The methodological quality of studies was assessed using the Cross-Sectional/Prevalence Study Quality Scale, as recommended by the US Agency for Healthcare Research and Quality (AHRQ) to provide a standard approach to methodological quality assessment in data interpretation [29]. The AHRQ scale is an 11-item tool using a score of 1 for items answered ‘*Yes*’ and 0 for items answered ‘*No*’, ‘*Unclear*’, or ‘*Not Applicable*’ [30]. Methodological quality of studies are graded based on the following scores: 0–3, *low quality*; 4–7, *moderate quality*; and 8–11, *high quality* [30]. One researcher undertook quality assessment, supervised by a second researcher.

### Data analysis

#### Descriptive statistics were used to analyse extracted quantitative study data

Given the heterogenous methodology, intervention types, and outcome measures, a meta-analysis was deemed inappropriate; therefore, a narrative synthesis approach was employed to evaluate the quantitative data from included studies. The narrative synthesis, based on thematic analysis, employed the following stages: familiarisation, initial coding, theme generation, refining themes with supporting evidence, and synthesis to summarise and assess the quantitative burden of diarrhoea outcomes in relation to different contexts and interventions [31,32].

## Results

**Figure 2.**
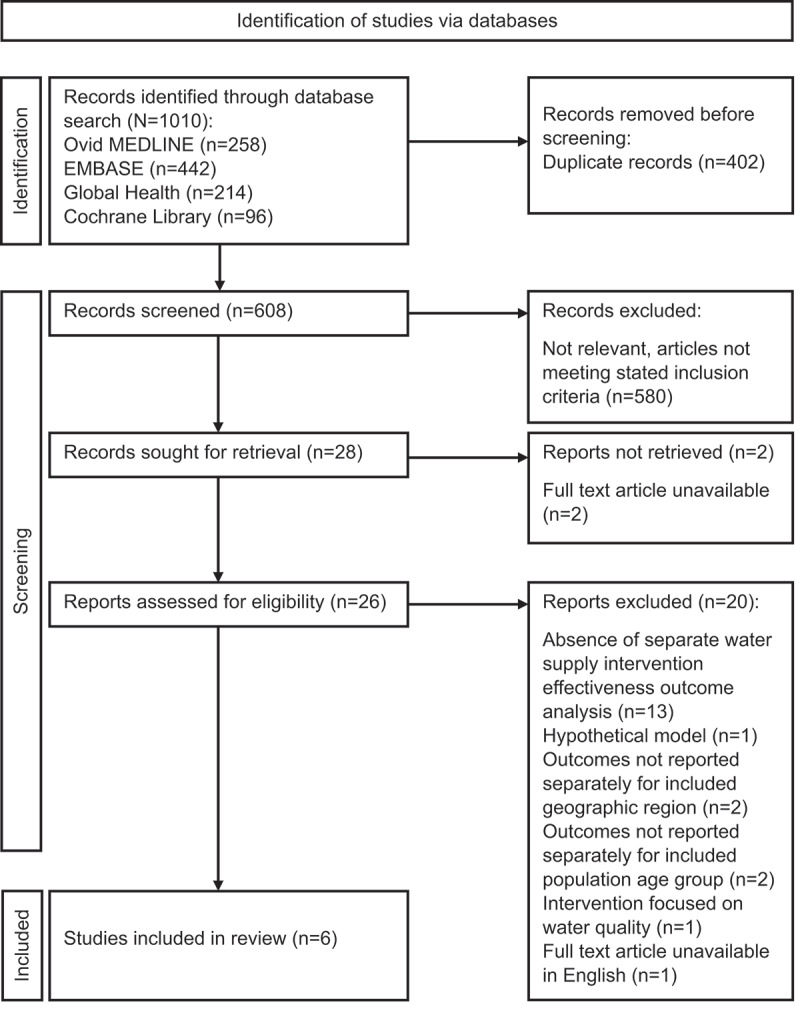
PRISMA flowchart of included studies.

The PRISMA flowchart of included studies is shown in Figure 2, and the full search string in Supplementary Appendix 3. The initial search yielded a total of 1010 studies, of which, screening identified six studies, which included over 110,000 datapoints from households and children <5 years. Of these, three studies reported diarrhoea prevalence in the context of communal water interventions, one study reported diarrhoeal morbidity based on hospitalisations in relation to a cash transfer-based program for water access, and two studies reported diarrhoeal mortality from a cash transfer-based program and country-level interventions for water access. All extracted study characteristics are reported in Table 1.

### Quantitative narrative synthesis: results of individual studies (Tables 1 and 2)

The six studies reported a range of outcomes from different water infrastructure intervention analyses. Communal provision (like piped water provided across a community area) of safe drinking water resulted in a decreased incidence of diarrhoeal disease. Specifically, a communal water system in Peru reduced diarrhoea incidence by 5% and a condominial water system by 37% in Peru and 33% in Guatemala. The benefits of communal provision were especially evident where reliable and uninterrupted service provision was present. Moreover, access to safe drinking water infrastructure via environmental health and cash-transfer program resulted in a decrease in the average morbidity rate from diarrhoea. Further to this, the same environmental health and cash-transfer program resulted in a decline in diarrhoeal-specific mortality rate. Additionally, increased household access to piped water resulted in a reduction in the under-five diarrhoeal-specific mortality rate from 23.3 per 1000 livebirths in 1980 to 0.8 per 1000 livebirths in 2015 in Peru.

### Thematic narrative synthesis

**Figure 3.**
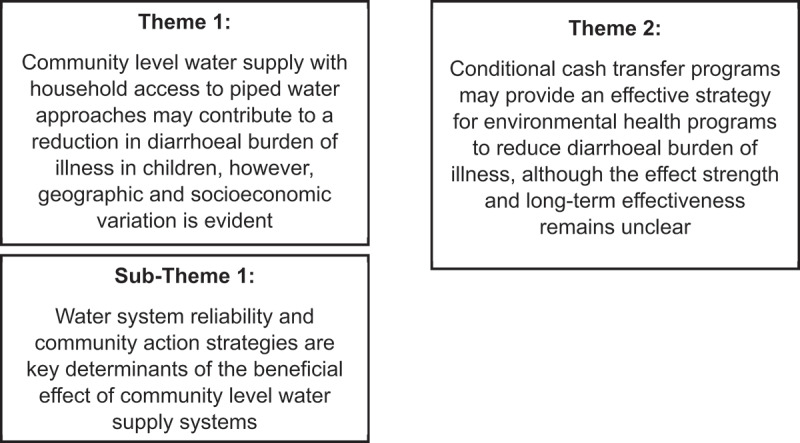
Identified themes on the effectiveness of water infrastructure interventions on the burden of diarrhoea in children <5 years in LAC.

The thematic analysis identified common themes within and across the six studies. Initial codes included community level water supply system characteristics, public water supply system, conditional cash transfer environmental health programs, household access to piped water, water system reliability and/or service interruption, reduction in diarrhoea outcome measure, no consistent or no beneficial effect on diarrhoea outcome measure. Overlapping and interconnected themes were identified, refined, and consolidated as the thematic analysis progressed to identify two overarching themes and one sub-theme (Figure 3).

### Theme 1: community level water supply with household access to piped water approaches may contribute to a reduction in diarrhoeal burden of illness in children, however, geographic and socioeconomic variation is evident

Systems based on communal implementation and management have been investigated in LAC. At this level, communal water supply with household access to piped water have shown effectiveness in different communities in LAC in contributing to a reduction in burden of diarrhoea in children (Tables 1 and 2). For instance, in Peru, a communal water supply system resulted in a decrease in diarrhoea prevalence between 0.098 and 0.163 (instrumental variable regressions) [33]. Additionally, in one remote community in Peru, the implementation of communal piped water infrastructure which provided inhouse connections to 76% of the study community resulted in a reduction in diarrhoeal disease of 46% for children <5 years in the service area [34]. However, this evidence is derived from a lower-quality study and results should be interpreted with caution (Supplementary Appendix 4). Moreover, the diarrhoeal-specific mortality rate in Peru reduced from 23.3 per 1000 livebirths in 1980 to 0.8 per 1000 livebirths in 2015, with household access to water identified as the intervention with the greatest impact on the reduction in burden of diarrhoeal disease [35]. Whilst an overall reduction in diarrhoea was reported, variations were identified from different geographic regions. For instance, diarrhoeal prevalence was reduced for the Sierra and Selva regions, whereas in the coastal region there was a null effect [33]. Moreover, socioeconomic factors contributed to variable effectiveness of communal water supply, with households of lower socioeconomic status experiencing higher incidence of diarrhoea whilst also being more likely to use communal water supplies [33].

**Table 2.** Burden of diarrhoea outcomes from empirical studies.

	Study sample	Diarrhoea case definition	Measure	Outcome
**Diarrhoeal prevalence**				
Calzada et al. *World Dev.*, 2021	Pooled cross-section of 84,600+ households and 26,200 children <age 5	Parental report of diarrhoea among child <age 5, within two weeks of survey	Linear probability of communal provision	Probability of communal provision −0.050 (*n*=22,436)
Hubbard et al. *J. Hyg. Environ.*, 2011	680 households with children <age 5	Number of cases of diarrhoea reported to the local clinic in Iquitos, Peru	Change in number of reported cases of diarrhoea	Decrease in incidence of diarrhoea by 37%
Trudeau et al. *IJPH*, 2018	7579 children from households with children <age 5	Reports of diarrhoea for children belonging to surveyed households	Change in reports of diarrhoea for children in surveyed households in Guatemala	Decrease in incidence of diarrhoea by 33%
**Diarrhoeal morbidity**				
de Souza et al. *PLoS ONE*, 2021	3467 municipalities from all regions; 38137 observations of children <age 5	ICD-10 A00-A04 and A06-A09	Access to water: IRR (95% CI) [p-value] with zero inflation (Brazil)	IRR=1.0073 (1.0062–1.0084) [<2e^−16^]
**Diarrhoeal mortality**				
de Souza et al. *BMC Public Health*, 2021	3467 municipalities from all regions; 38137 observations of children <age 5	ICD-10 A00-A04 and A06-A09	Access to water IRR (95% CI) [p-value] with zero inflation (Brazil)	Access to water >60%-≤85%: 0.6267 (0.4678–0.8396) [0.001737] Access to water >85%-≤100%: 0.5946 (0.4189–0.8440) [0.003624]
Huicho et al. *J. Glob. Health*, 2019	NR; Children <age 5	Under-five mortality and the cause-specific deaths	Under-five diarrhoea mortality (Peru) LiST analysis: Attribution of household access to piped water on the change in under-five diarrhoealspecific mortality rate (%)	23.3 per 1000 livebirths in 1980 to 0.8 per 1000 livebirths in 2015 30.4% in 1980–2015; 34.7% in 1980–2000; 26.2% in 2000–2015

CI=Confidence Interval; ICD=International Classification of Diseases; IRR=Incidence Rate Ratio; JASS=Juntas Administrativas de Servicios de Saneamiento; LiST=Lives Saved Tool; NR=Not Reported; SE=Standard Error.

### Sub-theme 1: water system reliability and community action strategies are key determinants of the beneficial effect of community level water supply systems

The effectiveness of communal approaches to increase access to safely managed drinking water, and in turn reduce the burden of diarrhoea in children, may depend on system reliability and community action (Tables 1 and 2). The effectiveness of a communal water system may therefore rely on the level of success of communal work by stakeholders, and good governance with consideration of social norms and traditions to facilitate communal water system success [33]. This highlights that tailored action strategies to the local community are required to maximise the effectiveness of the communal water system. Whilst access to safely managed drinking water through communal infrastructure approaches have demonstrated effectiveness in reducing the burden of diarrhoea in children <5 years in LAC, the reliability of the system may be a key moderating determinant (Tables 1 and 2). In Guatemala, evidence suggests that children in households with uninterrupted access to piped drinking water have a 33% reduction in diarrhoea incidence compared to children in households without access to piped water [36]. Further, evidence indicates that there was no difference in probability of diarrhoea incidence between children without access to piped drinking water and those with at least one day of service interruption [36]. This suggests that the public health benefits of access to piped water may be mediated by service reliability and community action strategies.

### Theme 2: conditional cash transfer programs may provide an effective strategy for environmental health programs to reduce diarrhoeal burden of illness, although the effect strength and long-term effectiveness remains unclear

Environmental health programs can be implemented in combination with conditional cash transfer programs to moderate and target access to services and monitor the resulting health impacts amongst identified population groups [37,38]. In Brazil, the effectiveness of a conditional cash transfer program in combination with access to water was assessed, and demonstrated that regions with high coverage of the target population and increased access to safe water resulted in lower rates of diarrhoeal mortality and morbidity amongst children <5 years [37,38]. However, given the variation in investment and coverage of the target population over time, there is limited evidence to support the overall effect strength of the conditional cash transfer program on diarrhoeal burden in children [37,38]. Moreover, the long-term effectiveness for this type of targeted infrastructure intervention to provide access to safely managed drinking water on reducing the burden of diarrhoea in children <5 years in LAC remains undetermined.

### Risk of bias (supplementary appendix 4)

Five studies were of moderate quality, of which, three received a score of 6/11, one received a score of 5/11, and another a score of 4/11. One report was deemed of low quality with a score of 2/11. All studies are limited by missing population and outcome data, the unblinded observational methodological approach, outcome assessment and outcome measurement bias, and a lack of empirical quality assurance.

## Discussion

### Main findings of this study

This study indicates that infrastructure interventions for the implementation of safely managed drinking water can provide public health benefits, evidenced by a reduction in burden of diarrhoea in children <5 years in LAC. Overall, the effectiveness of different intervention approaches to the supply of safely managed drinking water varies based on intervention type, setting, and management. In LAC, communal water systems were the most common intervention type studied. This SLR found that communal water systems based on the provision of household access to safely managed drinking water results in a reduction in diarrhoea prevalence in children <5 years in LAC. However, the efficacy of communal water supply on the burden of diarrhoea varies by geographic and socioeconomic factors. Moreover, in order to realise the full potential benefits of communal water system infrastructure, system reliability and community action strategies may be key determinants in moderating the beneficial impact on the burden of diarrhoea in children <5 years in LAC. Additionally, environmental health programs that employ conditional cash transfer to target provision to specific population groups may prove useful in a number of ways. This includes the provision of an effective approach to the implementation, management, and monitoring of benefits from infrastructure approaches to the provision of safely managed drinking water and a reduction in the burden of diarrhoea in children <5 years. However, the effect strength and long-term effectiveness of these programmes remains unclear.

### What is already known on this topic

Within the water sector, and considering the continually developing understanding of climate change impacts on the environment and human health, the UN Sustainable Development Goals have focused research, policy, and interventions on improving the safety and access to drinking water around the globe [1–3,8,9]. The UN, World Health Organization, World Bank, and United Nations Children’s Fund recommend a combination of different strategies related to access, quality, infrastructure, and financing in order to effectively achieve UN Sustainable Development Goal target 6.1 [9,39]. Previous SLRs in the sector have evaluated the effectiveness of water quality approaches on a reduction in burden of diarrhoea for children <5 years in low- and middle-income countries [23,24]. These SLRs have shown that improving water quality, through the use of water filters or safe storage devices at the household level, are effective at reducing the prevalence, morbidity, and mortality for children <5 years across low- and middle-income countries. However, these include limited evidence from LAC [23–25]. With respect to water access approaches, there is evidence to suggest that the development, implementation, and management of piped water systems in other geographic regions, such as Sub-Saharan Africa, are an effective approach to increasing the proportion of the population with access to safely managed drinking water [40]. In turn, access to safely managed drinking water sources in low- and middle-income countries are associated with improvements in child health primarily through a reduction in diarrhoeal disease [23,24,41]. Whilst there is evidence of effectiveness for water quality approaches, these recent SLRs have identified a gap in empirical evidence for water supply and access approaches in LAC [23,24]. Whilst piped water is widely recognised as an effective approach to improving access to safely managed drinking water globally, the feasibility of this approach in different geographic regions and for different population structures remains yet to be fully elucidated. Moreover, there is a gap in evidence for LAC where minimal research is available for the population across different geographic settings in LAC.

### What this study adds

This study provides a comprehensive review of the available empirical evidence on studies which investigate the effectiveness of water supply infrastructure interventions in LAC on burden of diarrhoea in children <5 years. This study has highlighted that communal water systems based on the implementation of piped water systems is the most common effective approach to improving access to safely managed drinking water and in turn reduces the burden of diarrhoeal disease in children in LAC, using the sustainable development goals as a theoretical basis. The strengths of this study are multi-fold: firstly, it is the first SLR of its kind that has systematically searched and analysed evidence from LAC on water infrastructure intervention effectiveness on burden of diarrhoea in children <5 years in LAC. Secondly, this SLR employed a quantitative narrative synthesis approach, which in addition to reporting the overall descriptive statistics from eligible studies, reported an analysis of the interrelated factors within the empirical evidence which provides a more detailed understanding of the effectiveness of different infrastructure interventions. This study provides a current overview of the empirical evidence for the effectiveness of water supply infrastructure interventions in LAC on diarrhoeal burden in children <5 years, indicating that communal water supply on the basis of piped water is the most common approach with demonstrated efficacy, however, this effectiveness may vary based on geographic and societal factors.

### Limitations of this study

The most significant limitation of this study is that the findings are based on observational unblinded studies of low to medium quality, which may result in biased findings. The observational nature of the empirical evidence may result in sampling and selection bias of specific subgroups of the population, limiting the representativeness of the evidence to other population groups and settings in LAC and broader settings. Moreover, the basis of the SLR is on publicly available literature published within four databases in English with research only available from 3 countries in LAC, and therefore, publication bias may impact the available evidence base. However, the use of a comprehensive and systematic search minimises this impact, whilst highlighting that future research is needed to examine the magnitude of water intervention effectiveness on burden of diarrhoea in children across different locations in LAC. In addition, given the limited empirical research available which utilised both different outcome measures and intervention types, limited the possibility for a meta-analysis to determine the overall effectiveness of interventions with respect to reducing the burden of diarrhoea in children <5 years. A further limitation is inherent to the use of a narrative synthesis approach which may introduce synthesis bias, although transparent reporting of the methodology reduces the potential impact of this on the findings [42]. This narrative synthesis provides an exploration and analysis of the factors important for interventions to improve access to safe drinking water with beneficial effects on burden of diarrhoea in children. Future research should aim to report consistent and comparable quantitative outcomes on intervention effectiveness to allow a future SLR with meta-analysis to determine the overall effect of water infrastructure interventions on reducing risk for diarrhoea in children in LAC. This, in turn, will inform future strategic and policy approaches to improve access to safe drinking water for public health benefits.

## Conclusion

In conclusion, this study provides novel evidence to suggest that in LAC, water infrastructure interventions that promote improved population access to safely managed water supply are effective at reducing the burden of diarrhoea in children <5 years. This study has demonstrated that water infrastructure interventions are effective in reducing the burden of diarrhoea in children <5 years in LAC. The study also reveals that various components of intervention strategies may enhance or limit the effectiveness of the intervention with respect to the impact on child health. Future studies – and a meta-analysis – are required to explore the effectiveness of different water infrastructure supply types in different settings across LAC to confirm the magnitude of efficacy of this approach to improving water access and reducing the associated high burden of diarrhoea in children. Water supply infrastructure intervention development, implementation, and management should consider a number of factors in the provision of safely managed drinking water: the provision of access at the household level, the reliability of the service, socioeconomic and demographic factors of the population, and the potential to combine interventions within broader environmental health or funding approaches.

## Supplementary Material

Supplemental Material
