# Supplementary material for: Effectiveness of infrastructural interventions to improve access to safe drinking water in Latin America and the Caribbean on the burden of diarrhoea in children <5 years: a systematic literature review and narrative synthesis
Source: Glob Health Action. 2025 Feb 14;18(1):2451610. doi: 10.1080/16549716.2025.2451610 (PMC11834799; doi:10.1080/16549716.2025.2451610)
Supplement: Supplemental Material [file ZGHA_A_2451610_SM2642.pdf]

# Appendices:

## Supplementary Appendix 1: PRISMA Reporting Guidelines

| Section and Topic | Item # | Checklist item | Location where item is reported |
|---|---|---|---|
| **TITLE** | | | |
| Title | 1 | Identify the report as a systematic review. | Page 1 |
| **ABSTRACT** | | | |
| Abstract | 2 | See the PRISMA 2020 for Abstracts checklist. | Pages 1-2 |
| **INTRODUCTION** | | | |
| Rationale | 3 | Describe the rationale for the review in the context of existing knowledge. | Pages 3-4; Figure 1 |
| Objectives | 4 | Provide an explicit statement of the objective(s) or question(s) the review addresses. | Pages 3-4 |
| **METHODS** | | | |
| Eligibility criteria | 5 | Specify the inclusion and exclusion criteria for the review and how studies were grouped for the syntheses. | Page 4 |
| Information sources | 6 | Specify all databases, registers, websites, organisations, reference lists and other sources searched or consulted to identify studies. Specify the date when each source was last searched or consulted. | Page 4; Suppl. 2 |
| Search strategy | 7 | Present the full search strategies for all databases, registers and websites, including any filters and limits used. | Suppl. 2 & 3 |
| Selection | 8 | Specify the methods used to decide whether a study met the | Page 4 |

| | | | |
|---|---|---|---|
| process | | inclusion criteria of the review, including how many reviewers screened each record and each report retrieved, whether they worked independently, and if applicable, details of automation tools used in the process. | |
| Data collection process | 9 | Specify the methods used to collect data from reports, including how many reviewers collected data from each report, whether they worked independently, any processes for obtaining or confirming data from study investigators, and if applicable, details of automation tools used in the process. | Pages 4-7; Table 1 |
| Data items | 10a | List and define all outcomes for which data were sought. Specify whether all results that were compatible with each outcome domain in each study were sought (e.g. for all measures, time points, analyses), and if not, the methods used to decide which results to collect. | Pages 4-7; Table 1 |
| | 10b | List and define all other variables for which data were sought (e.g. participant and intervention characteristics, funding sources). Describe any assumptions made about any missing or unclear information. | Pages 4-7; Table 1 |
| Study risk of bias assessment | 11 | Specify the methods used to assess risk of bias in the included studies, including details of the tool(s) used, how many reviewers assessed each study and whether they worked independently, and if applicable, details of automation tools used in the process. | Page 7 |
| Effect measures | 12 | Specify for each outcome the effect measure(s) (e.g. risk ratio, mean difference) used in the synthesis or presentation of results. | Pages 4-7; Table 1 |
| Synthesis methods | 13a | Describe the processes used to decide which studies were eligible for each synthesis (e.g. tabulating the study intervention characteristics and comparing against the planned groups for | Pages 4-7; Table 1 |

| | | | |
|---|---|---|---|
| | | each synthesis (item #5)). | |
| | 13b | Describe any methods required to prepare the data for presentation or synthesis, such as handling of missing summary statistics, or data conversions. | Not applicable |
| | 13c | Describe any methods used to tabulate or visually display results of individual studies and syntheses. | Page 7 |
| | 13d | Describe any methods used to synthesize results and provide a rationale for the choice(s). If meta-analysis was performed, describe the model(s), method(s) to identify the presence and extent of statistical heterogeneity, and software package(s) used. | Page 7 |
| | 13e | Describe any methods used to explore possible causes of heterogeneity among study results (e.g. subgroup analysis, meta-regression). | Not applicable |
| | 13f | Describe any sensitivity analyses conducted to assess robustness of the synthesized results. | Not applicable |
| Reporting bias assessment | 14 | Describe any methods used to assess risk of bias due to missing results in a synthesis (arising from reporting biases). | Not applicable |
| Certainty assessment | 15 | Describe any methods used to assess certainty (or confidence) in the body of evidence for an outcome. | Page 7 |
| **RESULTS** | | | |
| Study selection | 16a | Describe the results of the search and selection process, from the number of records identified in the search to the number of studies included in the review, ideally using a flow diagram. | Pages 7-8; Figure 2 |
| | 16b | Cite studies that might appear to meet the inclusion criteria, but which were excluded, and explain why they were excluded. | Pages 7-8; Figure 2 |

| | | | |
|---|---|---|---|
| Study characteristics | 17 | Cite each included study and present its characteristics. | Pages 4-7; Table 1 |
| Risk of bias in studies | 18 | Present assessments of risk of bias for each included study. | Page 14; Suppl. 4 |
| Results of individual studies | 19 | For all outcomes, present, for each study: (a) summary statistics for each group (where appropriate) and (b) an effect estimate and its precision (e.g. confidence/credible interval), ideally using structured tables or plots. | Pages 8-11; Table 2 |
| Results of syntheses | 20a | For each synthesis, briefly summarise the characteristics and risk of bias among contributing studies. | Pages 4-11; Tables 1 & 2, Figure 3 |
| | 20b | Present results of all statistical syntheses conducted. If meta-analysis was done, present for each the summary estimate and its precision (e.g. confidence/credible interval) and measures of statistical heterogeneity. If comparing groups, describe the direction of the effect. | Not applicable |
| | 20c | Present results of all investigations of possible causes of heterogeneity among study results. | Not applicable |
| | 20d | Present results of all sensitivity analyses conducted to assess the robustness of the synthesized results. | Not applicable |
| Reporting biases | 21 | Present assessments of risk of bias due to missing results (arising from reporting biases) for each synthesis assessed. | Not applicable |
| Certainty of evidence | 22 | Present assessments of certainty (or confidence) in the body of evidence for each outcome assessed. | Page 14; Suppl. 4 |
| **DISCUSSION** | | | |
| Discussion | 23a | Provide a general interpretation of the results in the context of | Pages |

| | | | |
|---|---|---|---|
| | | other evidence. | 14-17 |
| | 23b | Discuss any limitations of the evidence included in the review. | Pages 14-17 |
| | 23c | Discuss any limitations of the review processes used. | Pages 14-17 |
| | 23d | Discuss implications of the results for practice, policy, and future research. | Pages 14-17 |
| **OTHER INFORMATION** | | | |
| Registration and protocol | 24a | Provide registration information for the review, including register name and registration number, or state that the review was not registered. | Not applicable |
| | 24b | Indicate where the review protocol can be accessed, or state that a protocol was not prepared. | Not applicable |
| | 24c | Describe and explain any amendments to information provided at registration or in the protocol. | Not applicable |
| Support | 25 | Describe sources of financial or non-financial support for the review, and the role of the funders or sponsors in the review. | Not applicable |
| Competing interests | 26 | Declare any competing interests of review authors. | Not applicable |
| Availability of data, code and other materials | 27 | Report which of the following are publicly available and where they can be found: template data collection forms; data extracted from included studies; data used for all analyses; analytic code; any other materials used in the review. | Not applicable |

## Supplementary Appendix 2: Database specific search terms used in the systematic review

| | **Ovid Medline** | **EMBASE** | **Global Health** | **Cochrane Library** |
|---|---|---|---|---|

| | | | | |
|---|---|---|---|---|
| 1 | child* | child* | child* | child* |
| 2 | less than five | less than five | less than five | less than five |
| 3 | infant* | infant* | infant* | infant* |
| 4 | under-five | under-five | under-five | under-five |
| 5 | newborn* | newborn* | newborn* | newborn* |
| 6 | toddler* | toddler* | toddler* | toddler* |
| 7 | exp Child/ | exp child/ | exp children/ | MESH Child |
| 8 | exp Infant/ | exp newborn/ | exp infants/ | MESH Infant |
| 9 | 1 or 2 or 3 or 4 or 5 or 6 or 7 or 8 | 1 or 2 or 3 or 4 or 5 or 6 or 7 or 8 | 1 or 2 or 3 or 4 or 5 or 6 or 7 or 8 | 1 or 2 or 3 or 4 or 5 or 6 or 7 or 8 |
| 10 | Latin America and the Caribbean | Latin America and the Caribbean | Latin America and the Caribbean | Latin America and the Caribbean |
| 11 | LAC | LAC | LAC | LAC |
| 12 | Latin America* | Latin America* | Latin America* | Latin NEXT America* |
| 13 | Caribbean | Caribbean | Caribbean | Carribean |
| 14 | South America* | South America* | South America* | South NEXT America* |
| 15 | Central America* | Central America* | Central America* | Central NEXT America* |
| 16 | Americas | Americas | Americas | Americas |
| 17 | Antigua or Antiguan* or Antilles or Antillean Islands or Aruba or Aruban* or Barbuda or Barbudan* or Bahamas or Bahamian* or Caribbean or Cuba | Antigua or Antiguan* or Antilles or Antillean Islands or Aruba or Aruban* or Barbuda or Barbudan* or Bahamas or Bahamian* or Caribbean or Cuba | Antigua or Antiguan* or Antilles or Antillean Islands or Aruba or Aruban* or Barbuda or Barbudan* or Bahamas or Bahamian* or Caribbean or Cuba | Antigua or Antiguan* or Antilles or Antillean Islands or Aruba or Aruban* or Barbuda or Barbudan* or Bahamas or Bahamian* or Caribbean or Cuba |

| | | | | |
|---|---|---|---|---|
| | or Cuban or Curacao or Curacaoan* or Dominica or Dominican* or "Dominican Republic" or Grenada or Grenadian* or Grenadines or Guadeloupe or Guadeloupean* or Guadelouplan* or Haiti or Haitian or Jamaica* or Martinique or Martiniquais* or Martinican* or Nevis or Nevisian* or "Puerto Rico" or "Puerto Rican*" or "Saint Kitts" or Kittitian* or "Saint Lucia*" or "Saint Vincent" or Vincentian* or "Sint Maarten" or Sint Maartener* or Trinidad* or Trinidadian* or Tobago or | or Cuban or Curacao or Curacaoan* or Dominica or Dominican* or "Dominican Republic" or Grenada or Grenadian* or Grenadines or Guadeloupe or Guadeloupean* or Guadelouplan* or Haiti or Haitian or Jamaica* or Martinique or Martiniquais* or Martinican* or Nevis or Nevisian* or "Puerto Rico" or "Puerto Rican*" or "Saint Kitts" or Kittitian* or "Saint Lucia*" or "Saint Vincent" or Vincentian* or "Sint Maarten" or Sint Maartener* or Trinidad* or Trinidadian* or Tobago or | or Cuban or Curacao or Curacaoan* or Dominica or Dominican* or "Dominican Republic" or Grenada or Grenadian* or Grenadines or Guadeloupe or Guadeloupean* or Guadelouplan* or Haiti or Haitian or Jamaica* or Martinique or Martiniquais* or Martinican* or Nevis or Nevisian* or "Puerto Rico" or "Puerto Rican*" or "Saint Kitts" or Kittitian* or "Saint Lucia*" or "Saint Vincent" or Vincentian* or "Sint Maarten" or Sint Maartener* or Trinidad* or Trinidadian* or Tobago or | or Cuban or Curacao or Curacaoan* or Dominica or Dominican* or "Dominican Republic" or Grenada or Grenadian* or Grenadines or Guadeloupe or Guadeloupean* or Guadelouplan* or Haiti or Haitian or Jamaica* or Martinique or Martiniquais* or Martinican* or Nevis or Nevisian* or "Puerto Rico" or Puerto NEXT Rican* or "Saint Kitts" or Kittitian* or Saint NEXT Lucia* or "Saint Vincent" or Vincentian* or "Sint Maarten" or Sint Maartener* or Trinidad* or Trinidadian* or Tobago or |

| | | | | |
|---|---|---|---|---|
| | Tobagonian* or "Virgin Island*" or "West Indies" or "West Indian*" | Tobagonian* or "Virgin Island*" or "West Indies" or "West Indian*" | Tobagonian* or "Virgin Island*" or "West Indies" or "West Indian*" | Tobagonian* or Virgin NEXT Island* or "West Indies" or West NEXT Indian* |
| 18 | Belize or Belizean* or "Costa Rica" or "Costa Rican*" or "El Salvador" or Salvadoran* or Guatemala or Guatemalan* or Honduras or Honduran* or Mexico or Mexican* or Nicaragua or Nicaraguan* or Panama or Panamanian* or Argentina or Argentin* or Bolivia or Bolivian* or Brazil or Brazilian* or Chile or Chilean* or Colombia or Colombian* or Ecuador or Ecuadorian* or | Belize or Belizean* or "Costa Rica" or "Costa Rican*" or "El Salvador" or Salvadoran* or Guatemala or Guatemalan* or Honduras or Honduran* or Mexico or Mexican* or Nicaragua or Nicaraguan* or Panama or Panamanian* or Argentina or Argentin* or Bolivia or Bolivian* or Brazil or Brazilian* or Chile or Chilean* or Colombia or Colombian* or Ecuador or Ecuadorian* or | Belize or Belizean* or "Costa Rica" or "Costa Rican*" or "El Salvador" or Salvadoran* or Guatemala or Guatemalan* or Honduras or Honduran* or Mexico or Mexican* or Nicaragua or Nicaraguan* or Panama or Panamanian* or Argentina or Argentin* or Bolivia or Bolivian* or Brazil or Brazilian* or Chile or Chilean* or Colombia or Colombian* or Ecuador or Ecuadorian* or | Belize or Belizean* or "Costa Rica" or Costa NEXT Rican* or "El Salvador" or Salvadoran* or Guatemala or Guatemalan* or Honduras or Honduran* or Mexico or Mexican* or Nicaragua or Nicaraguan* or Panama or Panamanian* or Argentina or Argentin* or Bolivia or Bolivian* or Brazil or Brazilian* or Chile or Chilean* or Colombia or Colombian* or Ecuador or Ecuadorian* or |

| | | | | |
|---|---|---|---|---|
| | "French Guiana" or Guianan* or Guianese or Guyana or Guyanese or Paraguay or Paraguayan* or Peru or Peruvian* or Suriname or Surinamese or Uruguay or Uruguayan* or Venezuela or Venezuelan* | "French Guiana" or Guianan* or Guianese or Guyana or Guyanese or Paraguay or Paraguayan* or Peru or Peruvian* or Suriname or Surinamese or Uruguay or Uruguayan* or Venezuela or Venezuelan* | "French Guiana" or Guianan* or Guianese or Guyana or Guyanese or Paraguay or Paraguayan* or Peru or Peruvian* or Suriname or Surinamese or Uruguay or Uruguayan* or Venezuela or Venezuelan* | "French Guiana" or Guianan* or Guianese or Guyana or Guyanese or Paraguay or Paraguayan* or Peru or Peruvian* or Suriname or Surinamese or Uruguay or Uruguayan* or Venezuela or Venezuelan* |
| 19 | exp Latin America/ | exp South and Central America/ | exp Latin America/ | MESH Latin America/ |
| 20 | exp Caribbean Region/ | exp Caribbean/ | exp Cental America/ | MESH Caribbean Region/ |
| 21 | exp Central America/ | 10 or 11 or 12 or 13 or 14 or 15 or 16 or 17 or 18 or 19 or 20 | exp South America/ | MESH Central America/ |
| 22 | exp South America/ | water | exp Caribbean/ | MESH South America/ |
| 23 | 10 or 11 or 12 or 13 or 14 or 15 or 16 or 17 or 18 or 19 or 20 or 21 or 22 | new adj3 "water suppl*" | 10 or 11 or 12 or 13 or 14 or 15 or 16 or 17 or 18 or 19 or 20 or 21 or 22 | 10 or 11 or 12 or 13 or 14 or 15 or 16 or 17 or 18 or 19 or 20 or 21 or 22 |
| 24 | water | improved adj3 "water suppl*" | water | water |
| 25 | new adj3 "water suppl*" | pipe* | new adj3 "water suppl*" | new adj3 water NEXT suppl* |

| | | | | |
|---|---|---|---|---|
| 26 | improved adj3 "water suppl*" | borehole | improved adj3 "water suppl*" | improved adj3 water NEXT suppl* |
| 27 | pipe* | standpipe | pipe* | pipe* |
| 28 | borehole | tank* | borehole | borehole |
| 29 | standpipe | connect* | standpipe | standpipe |
| 30 | tank* | handpump* | tank* | tank* |
| 31 | "Sustainable Development Goal" adj3 "6.1" | infrastructur* | connect* | connect* |
| 32 | exp Drinking Water/ | capacit* | handpump* | handpump* |
| 33 | exp Water Supply/ | quantit* | infrastructur* | infrastructur* |
| 34 | exp Water Quality/ | program* adj3 evaluat* | capacit* | capacit* |
| 35 | connect* | "Sustainable Development Goal" adj3 "6.1" | quantit* | quantit* |
| 36 | handpump* | exp drinking water/ | program* adj3 evaluat* | program* adj3 evaluat* |
| 37 | infrastructur* | exp water supply/ | "Sustainable Development Goal" adj3 "6.1" | Sustainable Development Goal adj3 6.1 |
| 38 | capacit* | exp water quantity/ | exp drinking water/ | MESH Drinking Water/ |
| 39 | quantit* | exp water availability/ | exp water supply/ | MESH Water Supply/ |
| 40 | program* adj3 evaluat* | exp water management/ | exp water availability/ | MESH Water Quality/ |
| 41 | 24 or 25 or 26 or 27 or 28 or 29 or 30 or 31 or 32 or 33 or 34 | 22 or 23 or 24 or 25 or 26 or 27 or 28 or 29 or 30 or 31 or 32 | exp water distribution system/ | 24 or 25 or 26 or 27 or 28 or 29 or 30 or 31 or 32 or 33 or 34 |

| | | | | |
|---|---|---|---|---|
| | or 35 or 36 or 37 or 38 or 39 or 40 | or 33 or 34 or 35 or 36 or 37 or 38 or 39 or 40 | | or 35 or 36 or 37 or 38 or 39 or 40 or 41 |
| 42 | mortalit* | mortalit* | exp water resources/ | mortalit* |
| 43 | death* | death* | exp water security/ | death* |
| 44 | dying | dying | exp water rights/ | dying |
| 45 | loss of life | loss of life | exp water allocation/ | loss of life |
| 46 | fatal* | fatal* | exp water managament/ | fatal* |
| 47 | morbidit* | morbidit* | exp available water/ | morbidit* |
| 48 | prevalence | prevalence | exp available water capacity/ | prevalence |
| 49 | outcome* | outcome* | 24 or 25 or 26 or 27 or 28 or 29 or 30 or 31 or 32 or 33 or 34 or 35 or 36 or 37 or 38 or 39 or 40 or 41 or 42 or 43 or 44 or 45 or 46 or 47 or 48 | outcome* |
| 50 | rate* | rate* | mortalit* | rate* |
| 51 | risk* | risk* | death* | risk* |
| 52 | odds ratio | odds ratio | dying | odds ratio |
| 53 | incidence rate* | incidence rate* | loss of life | incidence NEXT rate* |
| 54 | IR | IR | fatal* | IR |
| 55 | relative risk | relative risk | morbidit* | relative risk |
| 56 | RR | RR | prevalence | RR |
| 57 | exp Mortality/ | exp childhood mortality/ | outcome* | MESH Mortality/ |
| 58 | exp Infant Death/ | exp infant mortality/ | rate* | MESH Infant Death/ |

| | | | | |
|---|---|---|---|---|
| 59 | exp Morbidity/ | exp child death/ | risk* | MESH Morbidity/ |
| 60 | exp Prevalence/ | exp mortality rate/ | odds ratio | MESH Prevalence/ |
| 61 | 42 or 43 or 44 or 45 or 46 or 47 or 48 or 49 or 50 or 51 or 52 or 53 or 54 or 55 or 56 or 57 or 58 or 59 or 60 | exp mortality risk/ | incidence rate* | 42 or 43 or 44 or 45 or 46 or 47 or 48 or 49 or 50 or 51 or 52 or 53 or 54 or 55 or 56 or 57 or 58 or 59 or 60 |
| 62 | diarrh?ea* | exp morbidity/ | IR | diarrh?ea* |
| 63 | exp Diarrhea/ | exp Prevalence/ | relative risk | MESH Diarrhea/ |
| 64 | 62 or 63 | 42 or 43 or 44 or 45 or 46 or 47 or 48 or 49 or 50 or 51 or 52 or 53 or 54 or 55 or 56 or 57 or 58 or 59 or 60 or 61 or 62 or 63 | RR | 62 or 63 |
| 65 | 9 and 23 and 41 and 61 and 64 | diarrh?ea* | exp mortality/ | 9 and 23 and 41 and 61 and 64 |
| 66 | limit 65 to english language and yr=2000-Current | exp diarrhea/ | exp morbidity/ | limit 65 to english language and yr=2000-2023 |
| 67 | | 65 or 66 | exp Prevalence/ | |
| 68 | | 9 and 21 and 41 and 64 and 67 | 50 or 51 or 52 or 53 or 54 or 55 or 56 or 57 or 58 or 59 or 60 or 61 or 62 or 63 or 64 or 65 or 66 or 67 | |

| | | | | |
|---|---|---|---|---|
| 69 | | limit 68 to english language and yr=2000-Current | diarrh?ea* | |
| 70 | | | exp diarrhoea/ | |
| 71 | | | 69 or 70 | |
| 72 | | | 9 and 23 and 49 and 68 and 71 | |
| 73 | | | limit 72 to english language and yr=2000-Current | |

*=Truncation; ?=Wildcard; IR=Incidence Rate; MESH=Medical Subject Heading; RR=Relative Risk.

## Supplementary Appendix 3: Full search string for the conducted SLR

**Database: Ovid MEDLINE(R) ALL <1946 to January 31, 2024>**

**Search Strategy:**

**1** child*.mp. [mp=title, book title, abstract, original title, name of substance word, subject heading word, floating sub-heading word, keyword heading word, organism supplementary concept word, protocol supplementary concept word, rare disease supplementary concept word, unique identifier, synonyms, population supplementary concept word, anatomy supplementary concept word] (2810130)

**2** less than five.mp. [mp=title, book title, abstract, original title, name of substance word, subject heading word, floating sub-heading word, keyword heading word, organism supplementary concept word, protocol supplementary concept word, rare disease supplementary concept word, unique identifier, synonyms, population supplementary concept word, anatomy supplementary concept word] (2925)

**3** infant*.mp. [mp=title, book title, abstract, original title, name of substance word, subject heading word, floating sub-heading word, keyword heading word, organism supplementary concept word, protocol supplementary concept word, rare disease supplementary concept word, unique identifier, synonyms, population supplementary concept word, anatomy supplementary concept word]

(1414403)

**4** under-five.mp. [mp=title, book title, abstract, original title, name of substance word, subject heading word, floating sub-heading word, keyword heading word, organism supplementary concept word, protocol supplementary concept word, rare disease supplementary concept word, unique identifier, synonyms, population supplementary concept word, anatomy supplementary concept word] (8211)

**5** newborn*.mp. [mp=title, book title, abstract, original title, name of substance word, subject heading word, floating sub-heading word, keyword heading word, organism supplementary concept word, protocol supplementary concept word, rare disease supplementary concept word, unique identifier, synonyms, population supplementary concept word, anatomy supplementary concept word] (846734)

**6** toddler*.mp. [mp=title, book title, abstract, original title, name of substance word, subject heading word, floating sub-heading word, keyword heading word, organism supplementary concept word, protocol supplementary concept word, rare disease supplementary concept word, unique identifier, synonyms, population supplementary concept word, anatomy supplementary concept word] (15144)

**7** exp Child/ (2185758)

**8** exp Infant/ (1266316)

**9** 1 or 2 or 3 or 4 or 5 or 6 or 7 or 8 (3557900)

**10** (Latin America and the Caribbean).mp. [mp=title, book title, abstract, original title, name of substance word, subject heading word, floating sub-heading word, keyword heading word, organism supplementary concept word, protocol supplementary concept word, rare disease supplementary concept word, unique identifier, synonyms, population supplementary concept word, anatomy supplementary concept word] (3303)

**11** LAC.mp. [mp=title, book title, abstract, original title, name of substance word, subject heading word, floating sub-heading word, keyword heading word, organism supplementary concept word, protocol supplementary concept word, rare disease supplementary concept word, unique identifier, synonyms, population supplementary concept word, anatomy supplementary concept word] (17956)

**12** Latin America*.mp. [mp=title, book title, abstract, original title, name of substance word, subject heading word, floating sub-heading word, keyword heading word, organism supplementary concept word, protocol supplementary concept word, rare disease supplementary concept word, unique identifier, synonyms, population supplementary concept word, anatomy supplementary concept word] (34343)

**13** Caribbean.mp. [mp=title, book title, abstract, original title, name of substance word, subject heading word, floating sub-heading word, keyword heading word, organism supplementary concept word, protocol supplementary concept word, rare disease supplementary concept word, unique identifier, synonyms, population supplementary concept word, anatomy supplementary concept word] (19535)

**14** South America*.mp. [mp=title, book title, abstract, original title, name of substance word, subject heading word, floating sub-heading word, keyword heading word, organism supplementary concept word, protocol supplementary concept word, rare disease supplementary concept word, unique identifier, synonyms, population supplementary concept word, anatomy supplementary concept word] (38918)

**15** Central America*.mp. [mp=title, book title, abstract, original title, name of substance word, subject heading word, floating sub-heading word, keyword heading word, organism supplementary concept word, protocol supplementary concept word, rare disease supplementary concept word, unique identifier, synonyms, population supplementary concept word, anatomy supplementary concept word] (9783)

**16** Americas.mp. [mp=title, book title, abstract, original title, name of substance word, subject heading word, floating sub-heading word, keyword heading word, organism supplementary concept word, protocol supplementary concept word, rare disease supplementary concept word, unique identifier, synonyms, population supplementary concept word, anatomy supplementary concept word] (29160)

**17** (Antigua or Antiguan* or Antilles or Antillean Islands or Aruba or Aruban* or Barbuda or Barbudan* or Bahamas or Bahamian* or Caribbean or Cuba or Cuban or Curacao or Curacaoan* or Dominica or Dominican* or "Dominican Republic" or Grenada or Grenadian* or Grenadines or Guadeloupe or Guadeloupean* or GuadeloupIan* or Haiti or Haitian or Jamaica* or Martinique or Martiniquais* or Martinican* or Nevis or Nevisian* or "Puerto Rico" or "Puerto Rican*" or "Saint Kitts" or Kittitian* or "Saint Lucia*" or "Saint Vincent" or Vincentian* or "Sint Maarten" or Sint Maartener* or Trinidad* or Trinidadian* or Tobago or Tobagonian* or "Virgin Island*" or "West Indies" or "West Indian*").mp. [mp=title, book title, abstract, original title, name of substance word, subject heading word, floating sub-heading word, keyword heading word, organism supplementary concept word, protocol supplementary concept word, rare disease supplementary concept word, unique identifier, synonyms,

population supplementary concept word, anatomy supplementary concept word] (59563)

**18** (Belize or Belizean* or "Costa Rica" or "Costa Rican*" or "El Salvador" or Salvadoran* or Guatemala or Guatemalan* or Honduras or Honduran* or Mexico or Mexican* or Nicaragua or Nicaraguan* or Panama or Panamanian* or Argentina or Argentin* or Bolivia or Bolivian* or Brazil or Brazilian* or Chile or Chilean* or Colombia or Colombian* or Ecuador or Ecuadorian* or "French Guiana" or Guianan* or Guianese or Guyana or Guyanese or Paraguay or Paraguayan* or Peru or Peruvian* or Suriname or Surinamese or Uruguay or Uruguayan* or Venezuela or Venezuelan*).mp. [mp=title, book title, abstract, original title, name of substance word, subject heading word, floating sub-heading word, keyword heading word, organism supplementary concept word, protocol supplementary concept word, rare disease supplementary concept word, unique identifier, synonyms, population supplementary concept word, anatomy supplementary concept word] (386021)

**19** exp Latin America/ (13447)

**20** exp Caribbean Region/ (33776)

**21** exp Central America/ (17397)

**22** exp South America/ (200842)

**23** 10 or 11 or 12 or 13 or 14 or 15 or 16 or 17 or 18 or 19 or 20 or 21 or 22 (508284)

**24** water.mp. [mp=title, book title, abstract, original title, name of substance word, subject heading word, floating sub-heading word, keyword heading word, organism supplementary concept word, protocol supplementary concept word, rare disease supplementary concept word, unique identifier, synonyms, population supplementary concept word, anatomy supplementary concept word] (1204857)

**25** (new adj3 "water suppl*").mp. [mp=title, book title, abstract, original title, name of substance word, subject heading word, floating sub-heading word, keyword heading word, organism supplementary concept word, protocol supplementary concept word, rare disease supplementary concept word, unique identifier, synonyms, population supplementary concept word, anatomy supplementary concept word] (103)

**26** (improved adj3 "water suppl*").mp. [mp=title, book title, abstract, original title, name of substance word, subject heading word, floating sub-heading word, keyword heading word, organism supplementary concept word, protocol supplementary concept word, rare disease supplementary concept word, unique identifier, synonyms, population supplementary concept word, anatomy

supplementary concept word] (183)

**27** pipe*.mp. [mp=title, book title, abstract, original title, name of substance word, subject heading word, floating sub-heading word, keyword heading word, organism supplementary concept word, protocol supplementary concept word, rare disease supplementary concept word, unique identifier, synonyms, population supplementary concept word, anatomy supplementary concept word] (196030)

**28** borehole.mp. [mp=title, book title, abstract, original title, name of substance word, subject heading word, floating sub-heading word, keyword heading word, organism supplementary concept word, protocol supplementary concept word, rare disease supplementary concept word, unique identifier, synonyms, population supplementary concept word, anatomy supplementary concept word] (1251)

**29** standpipe.mp. [mp=title, book title, abstract, original title, name of substance word, subject heading word, floating sub-heading word, keyword heading word, organism supplementary concept word, protocol supplementary concept word, rare disease supplementary concept word, unique identifier, synonyms, population supplementary concept word, anatomy supplementary concept word] (46)

**30** tank*.mp. [mp=title, book title, abstract, original title, name of substance word, subject heading word, floating sub-heading word, keyword heading word, organism supplementary concept word, protocol supplementary concept word, rare disease supplementary concept word, unique identifier, synonyms, population supplementary concept word, anatomy supplementary concept word] (23910)

**31** ("Sustainable Development Goal" adj3 "6.1").mp. [mp=title, book title, abstract, original title, name of substance word, subject heading word, floating sub-heading word, keyword heading word, organism supplementary concept word, protocol supplementary concept word, rare disease supplementary concept word, unique identifier, synonyms, population supplementary concept word, anatomy supplementary concept word] (15)

**32** exp Drinking Water/ (12526)

**33** exp Water Supply/ (35598)

**34** exp Water Quality/ (9710)

**35** connect*.mp. [mp=title, book title, abstract, original title, name of substance word, subject heading word, floating sub-heading word, keyword heading word, organism supplementary concept word, protocol supplementary concept word, rare disease supplementary concept word, unique identifier, synonyms, population supplementary concept word, anatomy supplementary concept word] (559498)

**36** handpump*.mp. [mp=title, book title, abstract, original title, name of substance word, subject heading word, floating sub-heading word, keyword heading word, organism supplementary concept word, protocol supplementary concept word, rare disease supplementary concept word, unique identifier, synonyms, population supplementary concept word, anatomy supplementary concept word] (61)

**37** infrastructur*.mp. [mp=title, book title, abstract, original title, name of substance word, subject heading word, floating sub-heading word, keyword heading word, organism supplementary concept word, protocol supplementary concept word, rare disease supplementary concept word, unique identifier, synonyms, population supplementary concept word, anatomy supplementary concept word] (62037)

**38** capacit*.mp. [mp=title, book title, abstract, original title, name of substance word, subject heading word, floating sub-heading word, keyword heading word, organism supplementary concept word, protocol supplementary concept word, rare disease supplementary concept word, unique identifier, synonyms, population supplementary concept word, anatomy supplementary concept word] (790617)

**39** quantit*.mp. [mp=title, book title, abstract, original title, name of substance word, subject heading word, floating sub-heading word, keyword heading word, organism supplementary concept word, protocol supplementary concept word, rare disease supplementary concept word, unique identifier, synonyms, population supplementary concept word, anatomy supplementary concept word] (1152781)

**40** (program* adj3 evaluat*).mp. [mp=title, book title, abstract, original title, name of substance word, subject heading word, floating sub-heading word, keyword heading word, organism supplementary concept word, protocol supplementary concept word, rare disease supplementary concept word, unique identifier, synonyms, population supplementary concept word, anatomy supplementary concept word] (91948)

**41** 24 or 25 or 26 or 27 or 28 or 29 or 30 or 31 or 32 or 33 or 34 or 35 or 36 or 37 or 38 or 39 or 40 (3785387)

**42** mortalit*.mp. [mp=title, book title, abstract, original title, name of substance word, subject heading word, floating sub-heading word, keyword heading word, organism supplementary concept word, protocol supplementary concept word, rare disease supplementary concept word, unique identifier, synonyms, population supplementary concept word, anatomy supplementary concept word]

(1444829)

**43** death*.mp. [mp=title, book title, abstract, original title, name of substance word, subject heading word, floating sub-heading word, keyword heading word, organism supplementary concept word, protocol supplementary concept word, rare disease supplementary concept word, unique identifier, synonyms, population supplementary concept word, anatomy supplementary concept word] (1155423)

**44** dying.mp. [mp=title, book title, abstract, original title, name of substance word, subject heading word, floating sub-heading word, keyword heading word, organism supplementary concept word, protocol supplementary concept word, rare disease supplementary concept word, unique identifier, synonyms, population supplementary concept word, anatomy supplementary concept word] (41586)

**45** loss of life.mp. [mp=title, book title, abstract, original title, name of substance word, subject heading word, floating sub-heading word, keyword heading word, organism supplementary concept word, protocol supplementary concept word, rare disease supplementary concept word, unique identifier, synonyms, population supplementary concept word, anatomy supplementary concept word] (1637)

**46** fatal*.mp. [mp=title, book title, abstract, original title, name of substance word, subject heading word, floating sub-heading word, keyword heading word, organism supplementary concept word, protocol supplementary concept word, rare disease supplementary concept word, unique identifier, synonyms, population supplementary concept word, anatomy supplementary concept word] (240884)

**47** morbidit*.mp. [mp=title, book title, abstract, original title, name of substance word, subject heading word, floating sub-heading word, keyword heading word, organism supplementary concept word, protocol supplementary concept word, rare disease supplementary concept word, unique identifier, synonyms, population supplementary concept word, anatomy supplementary concept word] (507751)

**48** prevalence.mp. [mp=title, book title, abstract, original title, name of substance word, subject heading word, floating sub-heading word, keyword heading word, organism supplementary concept word, protocol supplementary concept word, rare disease supplementary concept word, unique identifier, synonyms, population supplementary concept word, anatomy supplementary concept word] (913690)

**49** outcome*.mp. [mp=title, book title, abstract, original title, name of substance word, subject heading word, floating sub-heading word, keyword heading word, organism supplementary concept

word, protocol supplementary concept word, rare disease supplementary concept word, unique identifier, synonyms, population supplementary concept word, anatomy supplementary concept word] (3298352)

**50** rate*.mp. [mp=title, book title, abstract, original title, name of substance word, subject heading word, floating sub-heading word, keyword heading word, organism supplementary concept word, protocol supplementary concept word, rare disease supplementary concept word, unique identifier, synonyms, population supplementary concept word, anatomy supplementary concept word] (3836027)

**51** risk*.mp. [mp=title, book title, abstract, original title, name of substance word, subject heading word, floating sub-heading word, keyword heading word, organism supplementary concept word, protocol supplementary concept word, rare disease supplementary concept word, unique identifier, synonyms, population supplementary concept word, anatomy supplementary concept word] (3484539)

**52** odds ratio.mp. [mp=title, book title, abstract, original title, name of substance word, subject heading word, floating sub-heading word, keyword heading word, organism supplementary concept word, protocol supplementary concept word, rare disease supplementary concept word, unique identifier, synonyms, population supplementary concept word, anatomy supplementary concept word] (362707)

**53** incidence rate*.mp. [mp=title, book title, abstract, original title, name of substance word, subject heading word, floating sub-heading word, keyword heading word, organism supplementary concept word, protocol supplementary concept word, rare disease supplementary concept word, unique identifier, synonyms, population supplementary concept word, anatomy supplementary concept word] (74140)

**54** IR.mp. [mp=title, book title, abstract, original title, name of substance word, subject heading word, floating sub-heading word, keyword heading word, organism supplementary concept word, protocol supplementary concept word, rare disease supplementary concept word, unique identifier, synonyms, population supplementary concept word, anatomy supplementary concept word] (155905)

**55** relative risk.mp. [mp=title, book title, abstract, original title, name of substance word, subject heading word, floating sub-heading word, keyword heading word, organism supplementary concept word, protocol supplementary concept word, rare disease supplementary concept word, unique

identifier, synonyms, population supplementary concept word, anatomy supplementary concept word] (78428)

**56** RR.mp. [mp=title, book title, abstract, original title, name of substance word, subject heading word, floating sub-heading word, keyword heading word, organism supplementary concept word, protocol supplementary concept word, rare disease supplementary concept word, unique identifier, synonyms, population supplementary concept word, anatomy supplementary concept word] (97598)

**57** exp Mortality/ (425761)

**58** exp Infant Death/ (8230)

**59** exp Morbidity/ (658243)

**60** exp Prevalence/ (348955)

**61** 42 or 43 or 44 or 45 or 46 or 47 or 48 or 49 or 50 or 51 or 52 or 53 or 54 or 55 or 56 or 57 or 58 or 59 or 60 (10079482)

**62** diarrh?ea*.mp. [mp=title, book title, abstract, original title, name of substance word, subject heading word, floating sub-heading word, keyword heading word, organism supplementary concept word, protocol supplementary concept word, rare disease supplementary concept word, unique identifier, synonyms, population supplementary concept word, anatomy supplementary concept word] (144745)

**63** exp Diarrhea/ (58412)

**64** 62 or 63 (144745)

**65** 9 and 23 and 41 and 61 and 64 (457)

**66** limit 65 to (english language and yr="2000 -Current") (258)

---

**Database: Embase <1974 to 2024 January 31>**

**Search Strategy:**

**1** child*.mp. [mp=title, abstract, heading word, drug trade name, original title, device manufacturer, drug manufacturer, device trade name, keyword heading word, floating subheading word, candidate term word] (3256704)

**2** less than five.mp. [mp=title, abstract, heading word, drug trade name, original title, device manufacturer, drug manufacturer, device trade name, keyword heading word, floating subheading word, candidate term word] (4041)

**3** infant*.mp. [mp=title, abstract, heading word, drug trade name, original title, device manufacturer, drug manufacturer, device trade name, keyword heading word, floating subheading word, candidate term word] (1027652)

**4** under-five.mp. [mp=title, abstract, heading word, drug trade name, original title, device manufacturer, drug manufacturer, device trade name, keyword heading word, floating subheading word, candidate term word] (10258)

**5** newborn*.mp. [mp=title, abstract, heading word, drug trade name, original title, device manufacturer, drug manufacturer, device trade name, keyword heading word, floating subheading word, candidate term word] (755305)

**6** toddler*.mp. [mp=title, abstract, heading word, drug trade name, original title, device manufacturer, drug manufacturer, device trade name, keyword heading word, floating subheading word, candidate term word] (20118)

**7** exp child/ (3156280)

**8** exp newborn/ (613453)

**9** 1 or 2 or 3 or 4 or 5 or 6 or 7 or 8 (4041512)

**10** (Latin America and the Caribbean).mp. [mp=title, abstract, heading word, drug trade name, original title, device manufacturer, drug manufacturer, device trade name, keyword heading word, floating subheading word, candidate term word] (2996)

**11** LAC.mp. [mp=title, abstract, heading word, drug trade name, original title, device manufacturer, drug manufacturer, device trade name, keyword heading word, floating subheading word, candidate term word] (14760)

**12** Latin America*.mp. [mp=title, abstract, heading word, drug trade name, original title, device manufacturer, drug manufacturer, device trade name, keyword heading word, floating subheading word, candidate term word] (37159)

**13** Caribbean.mp. [mp=title, abstract, heading word, drug trade name, original title, device manufacturer, drug manufacturer, device trade name, keyword heading word, floating subheading word, candidate term word] (25285)

**14** South America*.mp. [mp=title, abstract, heading word, drug trade name, original title, device manufacturer, drug manufacturer, device trade name, keyword heading word, floating subheading word, candidate term word] (41156)

**15** Central America*.mp. [mp=title, abstract, heading word, drug trade name, original title, device manufacturer, drug manufacturer, device trade name, keyword heading word, floating subheading word, candidate term word] (34024)

**16** Americas.mp. [mp=title, abstract, heading word, drug trade name, original title, device manufacturer, drug manufacturer, device trade name, keyword heading word, floating subheading word, candidate term word] (15022)

**17** (Antigua or Antiguan* or Antilles or Antillean Islands or Aruba or Aruban* or Barbuda or Barbudan* or Bahamas or Bahamian* or Caribbean or Cuba or Cuban or Curacao or Curacaoan* or Dominica or Dominican* or "Dominican Republic" or Grenada or Grenadian* or Grenadines or Guadeloupe or Guadeloupean* or GuadeloupIan* or Haiti or Haitian or Jamaica* or Martinique or Martiniquais* or Martinican* or Nevis or Nevisian* or "Puerto Rico" or "Puerto Rican*" or "Saint Kitts" or Kittitian* or "Saint Lucia*" or "Saint Vincent" or Vincentian* or "Sint Maarten" or Sint Maartener* or Trinidad* or Trinidadian* or Tobago or Tobagonian* or "Virgin Island*" or "West Indies" or "West Indian*").mp. [mp=title, abstract, heading word, drug trade name, original title, device manufacturer, drug manufacturer, device trade name, keyword heading word, floating subheading word, candidate term word] (72526)

**18** (Belize or Belizean* or "Costa Rica" or "Costa Rican*" or "El Salvador" or Salvadoran* or Guatemala or Guatemalan* or Honduras or Honduran* or Mexico or Mexican* or Nicaragua or Nicaraguan* or Panama or Panamanian* or Argentina or Argentin* or Bolivia or Bolivian* or Brazil or Brazilian* or Chile or Chilean* or Colombia or Colombian* or Ecuador or Ecuadorian* or "French Guiana" or Guianan* or Guianese or Guyana or Guyanese or Paraguay or Paraguayan* or Peru or Peruvian* or Suriname or Surinamese or Uruguay or Uruguayan* or Venezuela or Venezuelan*).mp. [mp=title, abstract, heading word, drug trade name, original title, device manufacturer, drug manufacturer, device trade name, keyword heading word, floating subheading word, candidate term word] (476828)

**19** exp Caribbean/ (5672)

**20** south america/ or exp "south and central america"/ (299584)

**21** 10 or 11 or 12 or 13 or 14 or 15 or 16 or 17 or 18 or 19 or 20 (606744)

**22** water.mp. [mp=title, abstract, heading word, drug trade name, original title, device manufacturer, drug manufacturer, device trade name, keyword heading word, floating subheading word, candidate

term word] (1399425)

**23** (new adj3 "water suppl*").mp. [mp=title, abstract, heading word, drug trade name, original title, device manufacturer, drug manufacturer, device trade name, keyword heading word, floating subheading word, candidate term word] (137)

**24** (improved adj3 "water suppl*").mp. [mp=title, abstract, heading word, drug trade name, original title, device manufacturer, drug manufacturer, device trade name, keyword heading word, floating subheading word, candidate term word] (198)

**25** pipe*.mp. [mp=title, abstract, heading word, drug trade name, original title, device manufacturer, drug manufacturer, device trade name, keyword heading word, floating subheading word, candidate term word] (273208)

**26** borehole.mp. [mp=title, abstract, heading word, drug trade name, original title, device manufacturer, drug manufacturer, device trade name, keyword heading word, floating subheading word, candidate term word] (1359)

**27** standpipe.mp. [mp=title, abstract, heading word, drug trade name, original title, device manufacturer, drug manufacturer, device trade name, keyword heading word, floating subheading word, candidate term word] (91)

**28** tank*.mp. [mp=title, abstract, heading word, drug trade name, original title, device manufacturer, drug manufacturer, device trade name, keyword heading word, floating subheading word, candidate term word] (33270)

**29** connect*.mp. [mp=title, abstract, heading word, drug trade name, original title, device manufacturer, drug manufacturer, device trade name, keyword heading word, floating subheading word, candidate term word] (690577)

**30** handpump*.mp. [mp=title, abstract, heading word, drug trade name, original title, device manufacturer, drug manufacturer, device trade name, keyword heading word, floating subheading word, candidate term word] (71)

**31** infrastructur*.mp. [mp=title, abstract, heading word, drug trade name, original title, device manufacturer, drug manufacturer, device trade name, keyword heading word, floating subheading word, candidate term word] (73359)

**32** capacit*.mp. [mp=title, abstract, heading word, drug trade name, original title, device manufacturer, drug manufacturer, device trade name, keyword heading word, floating subheading

word, candidate term word] (1021157)

**33** quantit*.mp. [mp=title, abstract, heading word, drug trade name, original title, device manufacturer, drug manufacturer, device trade name, keyword heading word, floating subheading word, candidate term word] (1672725)

**34** (program* adj3 evaluat*).mp. [mp=title, abstract, heading word, drug trade name, original title, device manufacturer, drug manufacturer, device trade name, keyword heading word, floating subheading word, candidate term word] (54020)

**35** ("Sustainable Development Goal" adj3 "6.1").mp. [mp=title, abstract, heading word, drug trade name, original title, device manufacturer, drug manufacturer, device trade name, keyword heading word, floating subheading word, candidate term word] (18)

**36** exp drinking water/ (59858)

**37** exp water supply/ (47545)

**38** exp water quantity/ (209)

**39** exp water availability/ (3744)

**40** exp water management/ (217815)

**41** 22 or 23 or 24 or 25 or 26 or 27 or 28 or 29 or 30 or 31 or 32 or 33 or 34 or 35 or 36 or 37 or 38 or 39 or 40 (4822246)

**42** mortalit*.mp. [mp=title, abstract, heading word, drug trade name, original title, device manufacturer, drug manufacturer, device trade name, keyword heading word, floating subheading word, candidate term word] (1970379)

**43** death*.mp. [mp=title, abstract, heading word, drug trade name, original title, device manufacturer, drug manufacturer, device trade name, keyword heading word, floating subheading word, candidate term word] (1792527)

**44** dying.mp. [mp=title, abstract, heading word, drug trade name, original title, device manufacturer, drug manufacturer, device trade name, keyword heading word, floating subheading word, candidate term word] (59666)

**45** loss of life.mp. [mp=title, abstract, heading word, drug trade name, original title, device manufacturer, drug manufacturer, device trade name, keyword heading word, floating subheading word, candidate term word] (2009)

**46** fatal*.mp. [mp=title, abstract, heading word, drug trade name, original title, device manufacturer,

drug manufacturer, device trade name, keyword heading word, floating subheading word, candidate term word] (333930)

**47** morbidit*.mp. [mp=title, abstract, heading word, drug trade name, original title, device manufacturer, drug manufacturer, device trade name, keyword heading word, floating subheading word, candidate term word] (876433)

**48** prevalence.mp. [mp=title, abstract, heading word, drug trade name, original title, device manufacturer, drug manufacturer, device trade name, keyword heading word, floating subheading word, candidate term word] (1428109)

**49** outcome*.mp. [mp=title, abstract, heading word, drug trade name, original title, device manufacturer, drug manufacturer, device trade name, keyword heading word, floating subheading word, candidate term word] (4613536)

**50** rate*.mp. [mp=title, abstract, heading word, drug trade name, original title, device manufacturer, drug manufacturer, device trade name, keyword heading word, floating subheading word, candidate term word] (5464300)

**51** risk*.mp. [mp=title, abstract, heading word, drug trade name, original title, device manufacturer, drug manufacturer, device trade name, keyword heading word, floating subheading word, candidate term word] (5262174)

**52** odds ratio.mp. [mp=title, abstract, heading word, drug trade name, original title, device manufacturer, drug manufacturer, device trade name, keyword heading word, floating subheading word, candidate term word] (396269)

**53** incidence rate*.mp. [mp=title, abstract, heading word, drug trade name, original title, device manufacturer, drug manufacturer, device trade name, keyword heading word, floating subheading word, candidate term word] (108384)

**54** IR.mp. [mp=title, abstract, heading word, drug trade name, original title, device manufacturer, drug manufacturer, device trade name, keyword heading word, floating subheading word, candidate term word] (203219)

**55** relative risk.mp. [mp=title, abstract, heading word, drug trade name, original title, device manufacturer, drug manufacturer, device trade name, keyword heading word, floating subheading word, candidate term word] (100769)

**56** RR.mp. [mp=title, abstract, heading word, drug trade name, original title, device manufacturer,

drug manufacturer, device trade name, keyword heading word, floating subheading word, candidate term word] (149040)

**57** exp childhood mortality/ (14838)

**58** exp infant mortality/ (22260)

**59** exp child death/ (30938)

**60** exp mortality rate/ (143697)

**61** exp mortality risk/ (47778)

**62** exp morbidity/ (442978)

**63** exp Prevalence/ (1024874)

**64** 42 or 43 or 44 or 45 or 46 or 47 or 48 or 49 or 50 or 51 or 52 or 53 or 54 or 55 or 56 or 57 or 58 or 59 or 60 or 61 or 62 or 63 (14086983)

**65** diarrh?ea*.mp. [mp=title, abstract, heading word, drug trade name, original title, device manufacturer, drug manufacturer, device trade name, keyword heading word, floating subheading word, candidate term word] (366000)

**66** exp diarrhea/ (320120)

**67** 65 or 66 (372300)

**68** 9 and 21 and 41 and 64 and 67 (580)

**69** limit 68 to (english language and yr="2000 -Current") (442)

---

**Database: Global Health <1973 to 2024 Week 04>**

**Search Strategy:**

**1** child*.mp. [mp=abstract, title, original title, heading words, cabicodes words] (484902)

**2** less than five.mp. [mp=abstract, title, original title, heading words, cabicodes words] (1039)

**3** infant*.mp. [mp=abstract, title, original title, heading words, cabicodes words] (166054)

**4** under-five.mp. [mp=abstract, title, original title, heading words, cabicodes words] (6231)

**5** newborn*.mp. [mp=abstract, title, original title, heading words, cabicodes words] (37343)

**6** toddler*.mp. [mp=abstract, title, original title, heading words, cabicodes words] (3970)

**7** exp children/ (386850)

**8** exp infants/ (142864)

**9** 1 or 2 or 3 or 4 or 5 or 6 or 7 or 8 (595664)

**10** (Latin America and the Caribbean).mp. [mp=abstract, title, original title, heading words, cabicodes words] (11816)

**11** LAC.mp. [mp=abstract, title, original title, heading words, cabicodes words] (1553)

**12** Latin America*.mp. [mp=abstract, title, original title, heading words, cabicodes words] (204133)

**13** Caribbean.mp. [mp=abstract, title, original title, heading words, cabicodes words] (22666)

**14** South America*.mp. [mp=abstract, title, original title, heading words, cabicodes words] (168435)

**15** Central America*.mp. [mp=abstract, title, original title, heading words, cabicodes words] (12654)

**16** Americas.mp. [mp=abstract, title, original title, heading words, cabicodes words] (5930)

**17** (Antigua or Antiguan* or Antilles or Antillean Islands or Aruba or Aruban* or Barbuda or Barbudan* or Bahamas or Bahamian* or Caribbean or Cuba or Cuban or Curacao or Curacaoan* or Dominica or Dominican* or "Dominican Republic" or Grenada or Grenadian* or Grenadines or Guadeloupe or Guadeloupean* or GuadeloupIan* or Haiti or Haitian or Jamaica* or Martinique or Martiniquais* or Martinican* or Nevis or Nevisian* or "Puerto Rico" or "Puerto Rican*" or "Saint Kitts" or Kittitian* or "Saint Lucia*" or "Saint Vincent" or Vincentian* or "Sint Maarten" or Sint Maartener* or Trinidad* or Trinidadian* or Tobago or Tobagonian* or "Virgin Island*" or "West Indies" or "West Indian*").mp. [mp=abstract, title, original title, heading words, cabicodes words] (26446)

**18** (Belize or Belizean* or "Costa Rica" or "Costa Rican*" or "El Salvador" or Salvadoran* or Guatemala or Guatemalan* or Honduras or Honduran* or Mexico or Mexican* or Nicaragua or Nicaraguan* or Panama or Panamanian* or Argentina or Argentin* or Bolivia or Bolivian* or Brazil or Brazilian* or Chile or Chilean* or Colombia or Colombian* or Ecuador or Ecuadorian* or "French Guiana" or Guianan* or Guianese or Guyana or Guyanese or Paraguay or Paraguayan* or Peru or Peruvian* or Suriname or Surinamese or Uruguay or Uruguayan* or Venezuela or Venezuelan*).mp. [mp=abstract, title, original title, heading words, cabicodes words] (215337)

**19** exp Latin America/ (200797)

**20** exp Central America/ (11634)

**21** exp South America/ (163946)

**22** exp Caribbean/ (18868)

**23** 10 or 11 or 12 or 13 or 14 or 15 or 16 or 17 or 18 or 19 or 20 or 21 or 22 (252859)

**24** water.mp. [mp=abstract, title, original title, heading words, cabicodes words] (345886)

**25** (new adj3 "water suppl*").mp. [mp=abstract, title, original title, heading words, cabicodes words]

(137)

**26** (improved adj3 "water suppl*").mp. [mp=abstract, title, original title, heading words, cabicodes words] (251)

**27** pipe*.mp. [mp=abstract, title, original title, heading words, cabicodes words] (31542)

**28** borehole.mp. [mp=abstract, title, original title, heading words, cabicodes words] (646)

**29** standpipe.mp. [mp=abstract, title, original title, heading words, cabicodes words] (33)

**30** tank*.mp. [mp=abstract, title, original title, heading words, cabicodes words] (10369)

**31** connect*.mp. [mp=abstract, title, original title, heading words, cabicodes words] (44039)

**32** handpump*.mp. [mp=abstract, title, original title, heading words, cabicodes words] (77)

**33** infrastructur*.mp. [mp=abstract, title, original title, heading words, cabicodes words] (32120)

**34** capacit*.mp. [mp=abstract, title, original title, heading words, cabicodes words] (134038)

**35** quantit*.mp. [mp=abstract, title, original title, heading words, cabicodes words] (190341)

**36** (program* adj3 evaluat*).mp. [mp=abstract, title, original title, heading words, cabicodes words] (7145)

**37** ("Sustainable Development Goal" adj3 "6.1").mp. [mp=abstract, title, original title, heading words, cabicodes words] (23)

**38** exp drinking water/ (39779)

**39** exp water supply/ (15841)

**40** exp water availability/ (794)

**41** exp water distribution systems/ (2453)

**42** exp water resources/ (9150)

**43** exp water security/ (774)

**44** exp water rights/ (210)

**45** exp water allocation/ (254)

**46** exp water management/ (47652)

**47** exp available water/ (104)

**48** exp available water capacity/ (5)

**49** 24 or 25 or 26 or 27 or 28 or 29 or 30 or 31 or 32 or 33 or 34 or 35 or 36 or 37 or 38 or 39 or 40 or 41 or 42 or 43 or 44 or 45 or 46 or 47 or 48 (709949)

**50** mortalit*.mp. [mp=abstract, title, original title, heading words, cabicodes words] (262506)

**51** death*.mp. [mp=abstract, title, original title, heading words, cabicodes words] (293303)

**52** dying.mp. [mp=abstract, title, original title, heading words, cabicodes words] (5272)

**53** loss of life.mp. [mp=abstract, title, original title, heading words, cabicodes words] (494)

**54** fatal*.mp. [mp=abstract, title, original title, heading words, cabicodes words] (45653)

**55** morbidit*.mp. [mp=abstract, title, original title, heading words, cabicodes words] (98573)

**56** prevalence.mp. [mp=abstract, title, original title, heading words, cabicodes words] (399324)

**57** outcome*.mp. [mp=abstract, title, original title, heading words, cabicodes words] (330681)

**58** rate*.mp. [mp=abstract, title, original title, heading words, cabicodes words] (730919)

**59** risk*.mp. [mp=abstract, title, original title, heading words, cabicodes words] (853743)

**60** odds ratio.mp. [mp=abstract, title, original title, heading words, cabicodes words] (83155)

**61** incidence rate*.mp. [mp=abstract, title, original title, heading words, cabicodes words] (32129)

**62** IR.mp. [mp=abstract, title, original title, heading words, cabicodes words] (25397)

**63** relative risk.mp. [mp=abstract, title, original title, heading words, cabicodes words] (21301)

**64** RR.mp. [mp=abstract, title, original title, heading words, cabicodes words] (22182)

**65** exp mortality/ (180165)

**66** exp morbidity/ (40720)

**67** exp Prevalence/ (0)

**68** 50 or 51 or 52 or 53 or 54 or 55 or 56 or 57 or 58 or 59 or 60 or 61 or 62 or 63 or 64 or 65 or 66 or 67 (1803465)

**69** diarrh?ea*.mp. [mp=abstract, title, original title, heading words, cabicodes words] (59494)

**70** exp diarrhoea/ (38753)

**71** 69 or 70 (59494)

**72** 9 and 23 and 49 and 68 and 71 (345)

**73** limit 72 to (english language and yr="2000 -Current") (214)

---

Search Name: Cochrane Library FINAL Search_Version 2

Date Run: 01/02/2024 22:10:05

Comment: Final search: 01.02.24

ID Search Hits

#1 (child*):ti,ab,kw OR (less than five):ti,ab,kw OR (infant*):ti,ab,kw OR (under-five):ti,ab,kw OR (newborn*):ti,ab,kw 251881

#2 (toddler*):ti,ab,kw 2304

#3 MeSH descriptor: [Child] explode all trees 81197

#4 MeSH descriptor: [Infant] explode all trees 45750

#5 #1 OR #2 OR #3 OR #4 251994

#6 (Latin America and the Caribbean):ti,ab,kw OR (LAC):ti,ab,kw OR (Latin NEXT America*):ti,ab,kw OR (Caribbean):ti,ab,kw OR (South NEXT America*):ti,ab,kw 3433

#7 (Central NEXT America*):ti,ab,kw OR (Americas):ti,ab,kw OR (Antigua or Antiguan* or Antilles or Antillean Islands or Aruba or Aruban* or Barbuda or Barbudan* or Bahamas or Bahamian* or Caribbean or Cuba or Cuban or Curacao or Curacaoan* or Dominica or Dominican* or "Dominican Republic" or Grenada or Grenadian* or Grenadines or Guadeloupe or Guadeloupean* or GuadeloupIan* or Haiti or Haitian or Jamaica* or Martinique or Martiniquais* or Martinican* or Nevis or Nevisian* or "Puerto Rico" or Puerto NEXT Rican* or "Saint Kitts" or Kittitian* or Saint NEXT Lucia* or "Saint Vincent" or Vincentian* or "Sint Maarten" or Sint Maartener* or Trinidad* or Trinidadian* or Tobago or Tobagonian* or Virgin NEXT Island* or "West Indies" or West NEXT Indian*):ti,ab,kw OR (Belize or Belizean* or "Costa Rica" or Costa NEXT Rican* or "El Salvador" or Salvadoran* or Guatemala or Guatemalan* or Honduras or Honduran* or Mexico or Mexican* or Nicaragua or Nicaraguan* or Panama or Panamanian* or Argentina or Argentin* or Bolivia or Bolivian* or Brazil or Brazilian* or Chile or Chilean* or Colombia or Colombian* or Ecuador or Ecuadorian* or "French Guiana" or Guianan* or Guianese or Guyana or Guyanese or Paraguay or Paraguayan* or Peru or Peruvian* or Suriname or Surinamese or Uruguay or Uruguayan* or Venezuela or Venezuelan*):ti,ab,kw 19840

#8 MeSH descriptor: [Latin America] explode all trees 209

#9 MeSH descriptor: [Caribbean Region] explode all trees 559

#10 MeSH descriptor: [Central America] this term only 13

#11 MeSH descriptor: [South America] explode all trees 3960

#12 #6 OR #7 OR #8 OR #9 OR #10 OR #1121642

#13 (water):ti,ab,kw OR (new adj3 water NEXT suppl*):ti,ab,kw OR (improved adj3 water NEXT suppl*):ti,ab,kw OR (pipe*):ti,ab,kw OR (borehole):ti,ab,kw 52189

#14 (standpipe):ti,ab,kw OR (tank*):ti,ab,kw OR (connect*):ti,ab,kw OR (handpump*):ti,ab,kw OR (infrastructur*):ti,ab,kw 27482

#15 (capacit*):ti,ab,kw OR (quantit*):ti,ab,kw OR (program* adj3 evaluat*):ti,ab,kw OR (Sustainable Development Goal addj3 6.1):ti,ab,kw 96384

#16 MeSH descriptor: [Drinking Water] explode all trees 221

#17 MeSH descriptor: [Water Supply] explode all trees 254

#18 MeSH descriptor: [Water Quality] explode all trees 66

#19 #13 OR #14 OR #15 OR #16 OR #17 OR #18 168874

#20 (mortalit*):ti,ab,kw OR (death*):ti,ab,kw OR (dying):ti,ab,kw OR (loss of life):ti,ab,kw OR (fatal*):ti,ab,kw 195762

#21 (outcome*):ti,ab,kw OR (odds ratio):ti,ab,kw OR (incidence NEXT rate*):ti,ab,kw OR (IR):ti,ab,kw OR (relative risk):ti,ab,kw 838872

#22 (RR):ti,ab,kw OR (morbidit*):ti,ab,kw OR (prevalence):ti,ab,kw OR (rate*):ti,ab,kw OR (risk*):ti,ab,kw 738446

#23 MeSH descriptor: [Morbidity] explode all trees 21974

#24 MeSH descriptor: [Mortality] explode all trees 18841

#25 MeSH descriptor: [Infant Death] explode all trees129

#26 MeSH descriptor: [Prevalence] explode all trees 7381

#27 #20 OR #21 OR #22 OR #23 OR #24 OR #25 OR #26 1226251

#28 (diarrh?ea*):ti,ab,kw 34031

#29 MeSH descriptor: [Diarrhea] explode all trees 4429

#30 #28 OR #29 34031

#31 #5 AND #12 AND #19 AND #27 AND #30 with Cochrane Library publication date Between Jan 2000 and Jan 2024 96

**Supplementary Appendix 4: AHRQ quality assessment of included studies**

<u>Calzada, J. & Iranzo, S. World Development (2021).</u>

| AHRQ quality scale item | Yes | No | Unclear | NA |
|---|---|---|---|---|
| 1) Define the source of information (survey, record review) | X | | | |
| 2) List inclusion and exclusion criteria for exposed and unexposed subjects (cases and controls) or refer to previous publications | X | | | |
| 3) Indicate time period used for identifying patients | X | | | |
| 4) Indicate whether or not subjects were consecutive if not population-based | | | | X |
| 5) Indicate if evaluators of subjective components of study were masked to other aspects of the status of the participants | | | | X |
| 6) Describe any assessments undertaken for quality assurance purposes (e.g., test/retest of primary outcome measurements) | | | X | |
| 7) Explain any patient exclusions from analysis | X | | | |
| 8) Describe how confounding was assessed and/or controlled. | X | | | |

| | | | | |
|---|---|---|---|---|
| 9) If applicable, explain how missing data were handled in the analysis | X | | | |
| 10) Summarize patient response rates and completeness of data collection | | | X | |
| 11) Clarify what follow-up, if any, was expected and the percentage of patients for which incomplete data or follow-up was obtained | | | | X |

de Souza, A. A., Mingoti, S. A., Paes-Sousa, R., Heller, L. BMC Public Health (2021).

| AHRQ quality scale item | Yes | No | Unclear | NA |
|---|---|---|---|---|
| 1) Define the source of information (survey, record review) | X | | | |
| 2) List inclusion and exclusion criteria for exposed and unexposed subjects (cases and controls) or refer to previous publications | X | | | |
| 3) Indicate time period used for identifying patients | X | | | |
| 4) Indicate whether or not subjects were consecutive if not population-based | | | | X |
| 5) Indicate if evaluators of subjective components of study were masked to other aspects of the status of the participants | | | | X |
| 6) Describe any assessments undertaken for quality assurance purposes (e.g., test/retest of primary outcome measurements) | | | X | |

| | | | | |
|---|---|---|---|---|
| 7) Explain any patient exclusions from analysis | X | | | |
| 8) Describe how confounding was assessed and/or controlled. | X | | | |
| 9) If applicable, explain how missing data were handled in the analysis | | | | X |
| 10) Summarize patient response rates and completeness of data collection | X | | | |
| 11) Clarify what follow-up, if any, was expected and the percentage of patients for which incomplete data or follow-up was obtained | | | | X |

de Souza, A. A., Mingoti, S. A., Paes-Sousa, R., Heller, L. PLoS ONE (2021).

| AHRQ quality scale item | Yes | No | Unclear | NA |
|---|---|---|---|---|
| 1) Define the source of information (survey, record review) | X | | | |
| 2) List inclusion and exclusion criteria for exposed and unexposed subjects (cases and controls) or refer to previous publications | X | | | |
| 3) Indicate time period used for identifying patients | X | | | |
| 4) Indicate whether or not subjects were consecutive if not population-based | | | | X |
| 5) Indicate if evaluators of subjective components of study were masked to other aspects of the status of the participants | | | | X |

| | | | | |
|---|---|---|---|---|
| 6) Describe any assessments undertaken for quality assurance purposes (e.g., test/retest of primary outcome measurements) | | | X | |
| 7) Explain any patient exclusions from analysis | X | | | |
| 8) Describe how confounding was assessed and/or controlled. | X | | | |
| 9) If applicable, explain how missing data were handled in the analysis | | | | X |
| 10) Summarize patient response rates and completeness of data collection | X | | | |
| 11) Clarify what follow-up, if any, was expected and the percentage of patients for which incomplete data or follow-up was obtained | | | | X |

Hubbard, B., Sarisky, J., Gelting, R., Baffigo, V., Seminario, R., Centurion, C. International Journal of Hygiene & Environmental Health (2011).

| AHRQ quality scale item | Yes | No | Unclear | NA |
|---|---|---|---|---|
| 1) Define the source of information (survey, record review) | X | | | |
| 2) List inclusion and exclusion criteria for exposed and unexposed subjects (cases and controls) or refer to previous publications | | X | | |
| 3) Indicate time period used for identifying patients | | | X | |

| | | | | |
|---|---|---|---|---|
| 4) Indicate whether or not subjects were consecutive if not population-based | | | | X |
| 5) Indicate if evaluators of subjective components of study were masked to other aspects of the status of the participants | | | | X |
| 6) Describe any assessments undertaken for quality assurance purposes (e.g., test/retest of primary outcome measurements) | | | X | |
| 7) Explain any patient exclusions from analysis | | | X | |
| 8) Describe how confounding was assessed and/or controlled. | | | X | |
| 9) If applicable, explain how missing data were handled in the analysis | | | | X |
| 10) Summarize patient response rates and completeness of data collection | X | | | |
| 11) Clarify what follow-up, if any, was expected and the percentage of patients for which incomplete data or follow-up was obtained | | | | X |

Huicho, L., Tavera, M., Huayanay-Espinoza, C. A., Bejar-Diaz, M., Rivera-Ch, M., Tam, Y., Walker, N., Black, R. E. Journal of Global Health (2019).

| AHRQ quality scale item | Yes | No | Unclear | NA |
|---|---|---|---|---|
| 1) Define the source of information (survey, record review) | X | | | |

| | | | | |
|---|---|---|---|---|
| 2) List inclusion and exclusion criteria for exposed and unexposed subjects (cases and controls) or refer to previous publications | | X | | |
| 3) Indicate time period used for identifying patients | X | | | |
| 4) Indicate whether or not subjects were consecutive if not population-based | | | | X |
| 5) Indicate if evaluators of subjective components of study were masked to other aspects of the status of the participants | | | | X |
| 6) Describe any assessments undertaken for quality assurance purposes (e.g., test/retest of primary outcome measurements) | | | X | |
| 7) Explain any patient exclusions from analysis | | X | | |
| 8) Describe how confounding was assessed and/or controlled. | | X | | |
| 9) If applicable, explain how missing data were handled in the analysis | X | | | |
| 10) Summarize patient response rates and completeness of data collection | X | | | |
| 11) Clarify what follow-up, if any, was expected and the percentage of patients for which incomplete data or follow-up was obtained | | | | X |

Trudeau, J., Aksan, A. M., Vasquez, W. F. International Journal of Public Health (2018).

| AHRQ quality scale item | Yes | No | Unclear | NA |
|---|---|---|---|---|
| 1) Define the source of information (survey, record review) | X | | | |
| 2) List inclusion and exclusion criteria for exposed and unexposed subjects (cases and controls) or refer to previous publications | X | | | |
| 3) Indicate time period used for identifying patients | X | | | |
| 4) Indicate whether or not subjects were consecutive if not population-based | | | | X |
| 5) Indicate if evaluators of subjective components of study were masked to other aspects of the status of the participants | | | | X |
| 6) Describe any assessments undertaken for quality assurance purposes (e.g., test/retest of primary outcome measurements) | | | X | |
| 7) Explain any patient exclusions from analysis | X | | | |
| 8) Describe how confounding was assessed and/or controlled. | | | X | |
| 9) If applicable, explain how missing data were handled in the analysis | | | | X |
| 10) Summarize patient response rates and completeness of data collection | X | | | |
| 11) Clarify what follow-up, if any, was expected and the percentage of patients for which incomplete data or follow-up was obtained | | | | X |
